# Antibiotic-Resistant *Escherichia coli* and Sequence Type 131 in Fecal Colonization in Dogs in Taiwan

**DOI:** 10.3390/microorganisms8091439

**Published:** 2020-09-20

**Authors:** Jenn-Wei Chen, Han Hsiang Huang, Szu-Min Chang, Joy Scaria, Yu-Lung Chiu, Chih-Ming Chen, Wen-Chien Ko, Jiun-Ling Wang

**Affiliations:** 1 Department of Microbiology and Immunology, College of Medicine, National Cheng Kung University, Tainan 704, Taiwan; jc923@mail.ncku.edu.tw (J.-W.C.); s5nau3@gmail.com (S.-M.C.); 2 Department of Veterinary Medicine, National Chiayi University, Chiayi City 60054, Taiwan; hhuang@mail.ncyu.edu.tw (H.H.H.); handel737@yahoo.com.tw (Y.-L.C.); 3 Department of Veterinary and Biomedical Sciences, South Dakota State University, Brookings, SD 57007, USA; joy.scaria@sdstate.edu; 4 Department of Veterinary Medicine, National Chung Hsing University, Taichung 402, Taiwan; 5 Department of Nursing, Jenteh Junior College of Medicine, Nursing and Management, Miaoli 356, Taiwan; chioming2002@yahoo.com.tw; 6 Department of Internal Medicine, Tungs’ Taichung MetroHarbor Hospital, Taichung 435, Taiwan; 7 Department of Internal Medicine, National Cheng Kung University Hospital, Tainan 701, Taiwan; winston3415@gmail.com; 8 Department of Medicine, College of Medicine, National Cheng Kung University, Tainan 701, Taiwan

**Keywords:** dog, ST131, ESBL, fecal colonization, *Escherichia coli*

## Abstract

Background: Most drug-resistant *Escherichia coli* isolates in dogs come from diseased dogs. Prior to this study, the prevalence and risk factors of fecal carriage drug-resistant *E. coli* and epidemic clone sequence type (ST) 131 (including subtypes) isolates in dogs were unknown. Methods: Rectal swabs were used for *E. coli* isolation from 299 non-infectious dogs in a veterinary teaching hospital in Taiwan. Antibiotic resistance and multiplex PCR analyses of *E. coli* for major STs were performed. Result: There were 43.1% cefazolin-resistant, 22.1% fluoroquinolone-resistant, and 9.4% extended-spectrum beta-lactamase-producing *E. coli* in our cohort. In the phylogenetic study, B2 was the predominant group (30.1%). The cefazolin-resistant group and ciprofloxacin-resistant group had greater antibiotic exposure in the last 14 days (*p* < 0.05). The age, sex, and dietary habits of the antibiotic-resistant and -susceptible groups were similar. In the seven isolates of ST131 in fecal colonization, the most predominant subtypes were FimH41 and FimH22. Conclusion: Recent antibiotic exposure was related to the fecal carriage of antibiotic-resistant *E. coli* isolates. Three major subtypes (FimH41, H22, and H30) of ST131 can thus be found in fecal carriage in dogs in Taiwan.

## 1. Introduction

The close bond between humans and their dogs provides opportunities for the exchange of multidrug-resistant organisms. Moreover, the clinical infection of extended spectrum beta-lactamase (ESBL)-producing *Escherichia coli* in companion animals has been reported [1,2]. ESBL-producing *E. coli* have been reported since the late 1990s in companion animals in south-European countries and have now become widespread, with many nosocomial outbreaks in dogs in recent years [1,3]. Surveillance for antimicrobial resistance among bacteria isolated from dogs is useful for guiding antibiotic use when treating canine infections [2,4,5,6]. The urinary tract disease guidelines for dogs suggest that veterinarians should be aware of the pathogen and antimicrobial resistance trends among urinary pathogens isolated from patients in their clinic [7].

The prevalence of ESBL-producing *E. coli* was shown to be about 3% in clinical samples from companion animals in the US [8,9]. ESBL urinary tract infections (UTIs) in dogs have been reported in China and Switzerland in about 5% of samples [10,11]. In some laboratories (e.g., UK and China), there is an increasing percentage of extended-spectrum cephalosporin-resistant *E. coli* found in clinical isolates from companion animals [12,13].

Thus, beyond human isolates, the global emergence and spread of extraintestinal pathogenic *E. coli* O25-ST131 strains in ESBL isolates has also been found in companion animals [14,15]. According to the literature, ST131 can be found in clinical isolates from dogs in many countries, including the UK [12], the US [8,16], Canada [17], Japan [18,19], China [13], Germany [20,21], Australia [5,22], France [23], and Portugal [24,25,26]. Human–dog co-carriage was also demonstrated in several households [16,22,27]. Two large studies screening for ST131 in clinical samples in dogs determined about a 10% prevalence of ESBL-producing isolates [20] and 7% for fluoroquinolone-resistant *E. coli* [28]. In a study from Japan [19], ST131 constituted 36% of all clinical isolates of ESBL-producing *E. coli* infection among dogs and cats, and all subclones were FimH30. In comparison to FimH 30, fimH41 (clade A) and fimH22 (clade B) are usually antibiotic-susceptible alleles and are thought to be precursor subclones of FimH30. [29,30].

Few studies systematically collect rectal swabs or fecal carriage information for drug-resistant *E. coli* or ST131 in asymptomatic dogs. Moreover, the distribution of fimH type and other antibiotic-resistant genes in ST131 dog feces in Taiwan remains unclear. In this study, we investigate the prevalence of fluoroquinolone-resistant and beta-lactam-resistant *E. coli* in healthy dogs in Taiwan and determine if any risk factors, such as feeding habits or prior antibiotic use, are related to antibiotic-resistant *E. coli*. We also sought to study the subtypes and other antibiotic-resistant genes of the epidemic strain ST131 in asymptomatic dogs in Taiwan.

## 2. Materials and Methods

Rectal swabs from 299 dogs more than 6 months old were performed when the dogs visited the hospital for vaccinations and health exams. We collected information about sex, age, antibiotic use, and dietary habits from the owner. Dogs less than 6 months old or adopted less than 1 month prior to the study were excluded.

We used a BD^TM^ transport tube for anal/rectal swabbing. *E. coli* was isolated through a conventional method, and antibiotic resistance was determined by the disk diffusion method, including resistance of cefazolin, ciprofloxacin, ceftriaxone, gentamicin, and trimethoprim/sulfamethoxazole (TMP/SMZ). ESBL confirmation followed the recommendations of the Clinical and Laboratory Standards Institute (CLSI) by using a combined-disk test for ESBL production in Enterobacteriaceae. This test consists of measuring the growth-inhibitory zones around both the cefotaxime (CTX) and ceftazidime (CAZ) disks with or without clavulanate (CA) [31].

DNA was extracted with a MasterPure^TM^ complete DNA and RNA purification kit (Lucigen Corp., Middleton, WI, USA). Seven phylogroups were recognized (A, B1, B2, C, D, E, F) by multiplex PCR [32]. All PCR reactions were performed under the following conditions: 5 min at 95 °C for denaturation; 25 cycles of 30 s at 95 °C and 30 s at 59 °C (Quadruplex), 57 °C (Group C), or 55 °C (Group E); and 5 min at 72 °C for extension. The PCR product was measured with 2% agarose (Cyrusbioscience, Inc., New Taipei, Taiwan). The nine main *Escherichia coli* phylogroup B2 lineages involved in extra-intestinal infections were identified by the allele-specific PCR method [33]. All PCR reactions were performed under the following conditions: 4 min at 94 °C for denaturation and 25 cycles of 5 s at 94 °C, 20 s at 63 °C, and 5 min at 72 °C for extension. The PCR product was measured with 2% agarose (Cyrusbioscience, Inc., New Taipei, Taiwan).

In ESBL *E. coli* isolates, we performed ESBL gene screening including *bla*CTX-M, *bla*SHV, *bla*TEM, and *bla*OXA-1 by PCR using previously described methods [34]. In addition to *E. coli* colonization, we also screened for ST131 in some *E. coli* isolates from diseased dogs. If ST131 was found by the previous multiplex PCR, whole genome sequencing was performed. A genomic library was constructed using a Nextera XT DNA library preparation kit (Illumina, San Diego, CA, USA). We performed sequencing using an Illumina MiSeq platform with paired-end chemistry. All contigs were submitted to the CGE Finder Series (Centre for Genomic Epidemiology, Technical University of Denmark (DTU), https://cge.cbs.dtu.dk/services/).

The presence of resistance genes in the whole genome sequences and subtypes of the ST131 *E. coli* isolates was investigated by ResFinder and CHtyper [35].

## 3. Results

Among the 299 dogs, 26 dogs provided two isolates at collection. Fourteen dogs had no *E. coli* isolated when we performed the anal swab. We found 311 isolates for further antibiotic susceptibility tests and PCR studies, including a phylogenetic group and genotype study. In the antibiotic susceptibility test, there were 43.1% cefazolin-resistant and 22.1% fluoroquinolone-resistant *E. coli* colonies. Moreover, ESBL-producing *E. coli* fecal colonies were found in 9.4% of dogs. In the 28 isolates of ESBL-producing *E. coli*, ESBL gene can be found in *bla*SHV (n = 7), *bla*TEM (n = 2), *bla*OXA (n = 3), *bla*CTX-M group one (n = 19), group two (n = 8) and group nine (n = 9).

**Figure 1:**
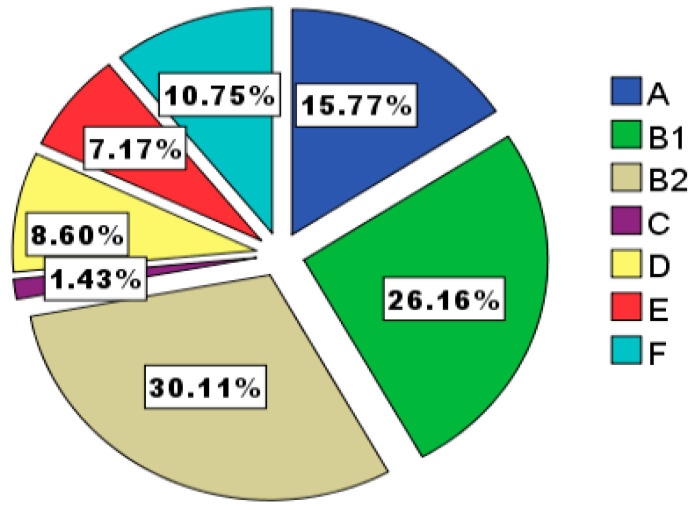
Phylogenetic group distribution (including group A, B1, B2, C, D, E, and F) of *E. coli* in the anal swabs of dogs.

In the phylogenetic study, 279 isolates were able to be grouped by PCR. The percentage of phylogenetic groups is shown in Figure 1. B2 was the predominant group (n = 84, 30.1%), followed by B1 (n = 73, 26.2%) and A (n = 44, 15.8%).

**Figure 2:**
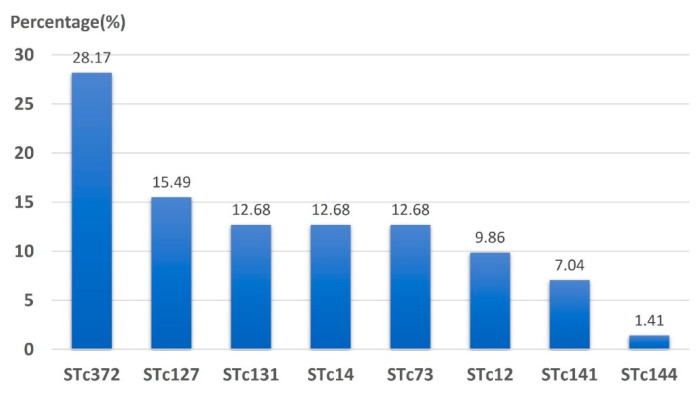
Sequence type, clonal complex (STc) distribution of phylogenetic group B2.

Among the 84 isolates of the B2 group, further clonal complexes could be found by multiplex PCR in 71 isolates. The distributions of common sequence type (ST) complexes are shown in Figure 2. The three most predominant ST complexes were STc372, followed by STc127 and STc131.

In the analysis of the risk factors for ESBL-producing *E. coli*, the food habits of the animals were not different. However, the ESBL-producing group had more antibiotic exposure in the last 14 days prior to the study (39.3% vs. 13.7%, *p* < 0.05) (Table 1). The cefazolin-resistant *E. coli* group also had higher antibiotic exposure than cefazolin-susceptible *E. coli* (26.4% vs. 8.0%, *p* < 0.05) (Table 1). In the comparison between fluoroquinolone-resistant *E. coli* and fluoroquinolone-susceptible *E. coli*, the trend of antibiotic exposure in the last 14 days was similar (40.9% vs. 9.0%, *p* < 0.05). The dietary habits were similar between the fluroquinolone-resistant and fluoroquinolone-susceptible groups (Table 1). Different antibiotic exposure histories were not found in the phylogenetic B2 and non-B2 groups (Table 2).

A further whole genome analysis was performed on 10 ST131 isolates, including seven isolates of fecal colonization and three isolates of clinical infection. The FimH types and antibiotic-resistant genes found by Res finder and the antibiotic resistance phenotypes found via the disk diffusion method are shown in Table 3.

Among the seven isolates of ST131 in the fecal colonies, the most predominant subtypes were FimH41 (42.8%, n = 3) and FimH22 (42.8%, n = 3). Moreover, FimH30 was found in only one (14.3%) of the fecal isolates but in 100% of the three clinical isolates from dogs with infections. ESBL enzymes in the fecal colonies included CTX-M15 (n = 1), CTX-M55 (n = 1), and CTX-M65 (n = 1). All of the infection isolates were caused by CTX-M15-producing *E. coli* isolates.

## 4. Discussion

This is the first study on *E. coli* fecal colonization with a risk factor analysis in Taiwan. We found that recent antibiotic exposure was associated with cefazolin-resistant, fluoroquinolone-resistant, and ESBL-producing *E. coli*. Nearly half of the *E. coli* isolates were resistant to cefazolin, about 20% were resistant to fluoroquinolone, and about 10% were ESBL-producing. Among healthy dogs in France and Spain, the rate of third generation cephalosporin resistance and fluroquinolone resistance was about 18% according to the anal swab [36]. In Pakistan, the percentage of ESBL-producing *E. coli* isolates in dog feces was shown to be as high as 80% [37], while data from the US showed a prevalence of less than 10% in dogs [38]. Our study illustrates the challenges of emerging drug-resistant pathogens in companion animals.

The predominant B2 phylogenetic clonal complexes in our cohort were STc372 and STc127. The predominant role of ST372 in this study was similar to that of the clinical isolates from dogs in the US [39] and France [40]. In Israel, ST127 was found in ESBL *E. coli* from a petting zoo [41] and in infected cats from Australia [42]. In another human study in the US [43], ST127 was significantly more frequently recovered from UTI samples and was the second most common ST type in young woman with UTIs in Canada [44]. ST372 was also the second most prevalent clone and the highest patient-to-patient transmission clone in a study in rehabilitation wards in Israel [45].

Similar to previous studies, the risk factors of underlying disease conditions and the use of prior antimicrobial agents were the primary risks associated with a UTI presenting MDR (multi-drug-resistant) *E. coli* in dogs [5]. In our ESBL *E.coli* isolates, the most prevalent one was found to be CTX-M group one. In a study on the ESBL genotype in diseased cats and dogs in China, CTX-M-65 and CTX-M-15 were the most predominant CTX-M enzyme types among *E. coli* isolates [13]. Furthermore, in the UK and Japan, CTX-M-15, CTX-M-14, and CTX-M-55 were also found to be prevalent in clinical isolates from companion animals [12,18]. ST131 CTX-M-15-producing *E. coli* strains are common in the clinical isolates of companion animals [20]. In addition to clinical isolates from infected animals, CTX-M15 was also found in the asymptomatic fecal carriers of ST131 in our cohort. CTX-M-15, which spreads pandemically among humans, was only detected in 15% of companion animals [46]. Other CTX-M types (CTX-M-55 and -65) were also found in our fecal colonized isolates. In a previous study of households in Sweden, humans carried identical strain of ESBL *E. coli* to the isolates found in household dogs, indicating a transfer between humans and dogs [47]

*E. coli* ST131 adapt to be so successful and is now a major global health issue in both human and companion animals [48]. This may be explained by high antimicrobial resistance without any fitness cost [48]. In our cohort, ST131 was found in about 13% of B2 isolates and about 2.3% of all healthy dogs. In another human fecal carrier study in Taiwan, ST131 was found in 3.0% in healthy adults [49]. The percentage of ST131 carriage in the fecal carrier seems similar between humans and dogs. In a study from dogs and cats admitted to a veterinary teaching hospital in Taiwan, ST131 was the second most common ST (15.4%) in isolates with ESBL phenotype [50]. In a previous study of household pets, the index dog’s urinary tract inflection strain was found to be a prevalent human-associated variant of *E. coli* ST131. This suggest a host-to-host transmission of ST131 among household pets [16]. Similar to the findings for humans, the fecal *E. coli* colonization of ST131 in our dog cohort demonstrated that subtype FimH22 and FimH41 strains have the ability to colonize the gut. Moreover, the H30 strains displayed traits that allow extra-intestinal infection [51]. A recent study showed that H22 accounts for nearly all ST131 meat isolates and for about 10% of ST131 clinical isolates [52]. In a healthy human fecal colonization study, O16-ST131 with FimH41 isolates was found to be dominant [53]. In our three isolates of Fim41, two (66%) were serotype O16 (data not shown).

## 5. Conclusions

In our *E. coli* fecal carriage study, the common STs circulating among humans were also found in dog isolates. The risk factor analysis similarly showed that antibiotic use leads to multidrug-resistant *E. coli* colonization. Ultimately, three important subclones of the epidemic clones ST131 (Fim30, Fim22, and Fim41) were found in canine fecal carriage in Taiwan.

## Figures and Tables

**Table 1:** Risk factors of antimicrobial-resistant *E. coli* in the fecal carriage of dogs.

	ESBL-Producing *E. coli*	Non-ESBL-Producing *E. coli*	*p*
N	28	271	
Age, years	8.5(4.7)	9.1(4.7)	0.562
Sex	14.0(50.0)	140(51.7)	0.867
Commercial pet food	9(32.1)	66(24.4)	0.365
Human food use	17(60.7)	178(65.7)	0.599
Recent use of antimicrobial agents	11(39.3)	37(13.7)	<0.001
	**Cefazolin** **-resistant**	**Cefazolin** **-susceptible**	
N	129	170	
Age	9.1(4.8)	9.0(4.7)	0.819
Sex	65(50.4)	89(52.4)	0.736
Commercial pet food	33(25.6)	42(24.7)	0.863
Human food use	85(65.9)	110(64.7)	0.831
Recent use of antimicrobial agents	34(26.4)	14(8.2)	<0.001
	**Ciprofloxacin-** **resistant**	**Ciprofloxacin** **-susceptible**	
N	66	233	
Age	9.0(4.5)	9.0(4.8)	0.989
Sex	36(54.5)	118(50.6)	0.576
Commercial pet food	18(27.3)	57(24.5)	0.642
Human food use	43(65.2)	152(65.2)	0.990
Recent use of antimicrobial agents	27(40.9)	21(9.0)	<0.001

Note: Some dogs had two *E. coli* isolates in their fecal specimen, and some did not provide any *E. coli*; ESBL extended-spectrum β-lactamases.

**Table 2:** Risk factors of *E. coli* fecal carriage of phylogenetic B2.

	Phylogenetic B2 Carrier	Non-Phylogenetic B2 Carrier	*p*
N	79	220	
Age	10.1(4.9)	8. 6(4.6)	0.023
Sex	41(51.9)	113(51.4)	0.935
commercial pet food	20(25.3)	55(25.0)	0.956
human food use	55(69.6)	140(63.6)	0.338
recent use of antimicrobial agents	13(16.5)	35(15.9)	0.910

**Table 3:** The antibiotic resistance profiles (genes and phenotypes) of 7 fecal carriages of ST131 isolates and 3 clinical isolates from diseased dogs.

FimH Type	Aminoglycoside R Gene and Phenotype		TMP/SMZ R Gene and Phenotype		Cephalosporin R Gene and Phenotype (Cefazolin, Ceftriaxone, ESBL)		Quinolone R Gene and Phenotype	
**Fecal Carriage**								
22	aac(3)-IId-like, aadA12-like	R	sul2-like, sul3, dfrA12	R	*bla*_CTX-M-65_, *bla*_TEM-1B-like_	R, R, +	QnrS1-like	R
22	nd	S	nd	S	nd	S, S, -	nd	S
41 *	aac(3)-IId-like	R	sul2, dfrA14-like	R	*bla*_CMY-2_, *bla*_CTX-M-55_	R, R, +	QnrS1	S
22 ^#^	aac(3)-IId-like, strA, strB-like	R	sul2-like	S	nd	S, S, -	nd	R
41 ^#^	aac(3)-IId-like, strA, strB-like	R	sul2-like	S	*bla*_TEM-1B_	S, S, -	N	R
30	aac(3)-IIa-like, aadA5	R	sul1, dfrA17	R	*bla*_CTX-M-15_	R, R, +	aac(6’)Ib-cr	R
41	aadA5, strA, strB-like	S	sul1, sul2-like, dfrA17	R	*bla*_TEM-1B-like_	S, S, -	nd	R
**Clinical isolates from diseased dogs**								
30	aac(6’)-Ib-cr	S	nd	S	*bla*_CTX-M-15_	R, R, +	aac(6’)-Ib-cr	S
30	aac(6’)-Ib-cr	S	nd	S	*bla*_CTX-M-15_	R, R, +	aac(6’)-Ib-cr	R
30	nd	S	nd	S	nd	R, R, -	nd	R

R: resistant S: susceptible; +: ESBL phenotype positive; -: ESBL phenotype negative; nd: not detected; #: recent antimicrobial agent use history, including cephalexin* and amoxicillin/clavunate#.
